# When push comes to shove - RNA polymerase and DNA-bound protein roadblocks

**DOI:** 10.1007/s12551-023-01064-7

**Published:** 2023-06-10

**Authors:** Nan Hao, Alana J. Donnelly, Ian B. Dodd, Keith E. Shearwin

**Affiliations:** Department of Molecular and Biomedical Science, School of Biological Sciences, The University of Adelaide, Adelaide, SA 5005 Australia

**Keywords:** RNA polymerase, Transcriptional roadblocking, Transcriptional interference, DNA binding kinetics, CRISPR

## Abstract

In recent years, transcriptional roadblocking has emerged as a crucial regulatory mechanism in gene expression, whereby other DNA-bound obstacles can block the progression of transcribing RNA polymerase (RNAP), leading to RNAP pausing and ultimately dissociation from the DNA template. In this review, we discuss the mechanisms by which transcriptional roadblocks can impede RNAP progression, as well as how RNAP can overcome these obstacles to continue transcription. We examine different DNA-binding proteins involved in transcriptional roadblocking and their biophysical properties that determine their effectiveness in blocking RNAP progression. The catalytically dead CRISPR-Cas (dCas) protein is used as an example of an engineered programmable roadblock, and the current literature in understanding the polarity of dCas roadblocking is also discussed. Finally, we delve into a stochastic model of transcriptional roadblocking and highlight the importance of transcription factor binding kinetics and its resistance to dislodgement by an elongating RNAP in determining the strength of a roadblock.

## Introduction

Gene expression in both prokaryotes and eukaryotes involves sophisticated regulatory mechanisms, with much of this regulation occurring at the transcription level. Transcription is a multistage process that involves the binding of RNA polymerase (RNAP) to a promoter region on the DNA, followed by the unwinding of the double helix. Once the DNA is unwound, RNAP travels along the DNA to synthesize an RNA molecule until it reaches the end of the gene or transcription unit, where it is terminated, and the newly synthesized RNA molecule is released. Transcriptional control takes place at all three stages of this process—initiation, elongation and termination. In this review, we will focus on the regulation that occurs during transcription elongation, where RNAP must navigate various obstacles while traveling along the DNA.

During transcription elongation, RNAP may encounter various obstacles that can impede its progression. These obstacles can include DNA pause sites, which temporarily halt the progression of RNAP, thereby altering the rate of RNA synthesis (Mayer et al. 2017), as well as DNA lesions on the transcribed DNA caused by UV damage or chemical alteration (Gregersen and Svejstrup 2018; Nadon et al. 2022; Strobel et al. 2020). Additionally, RNAP may encounter static or mobile protein roadblocks that hinder its progression. Mobile protein blocks are formed by proteins that move along the same DNA, including other RNA polymerases and DNA polymerases required for DNA metabolism (Helmrich et al. 2011; Le and Wang 2018; Shearwin et al. 2005; Wang et al. 2023). On the other hand, static DNA-bound obstacles are formed by proteins such as transcription factors that bind to specific DNA sites or by non-specific DNA-binding proteins (Choi and Saier 2005; Dole et al. 2004; He and Zalkin 1992; Lewis et al. 2008). These static DNA-bound obstacles can pose a physical barrier that slows down or impedes RNAP progression, thereby affecting gene expression (Epstein et al. 2003; Hao et al. 2014). In the following section, we will discuss the details of these static roadblocks and their effects on transcription elongation.

## Transcriptional roadblocking: mechanisms and consequences

**Fig. 1:**
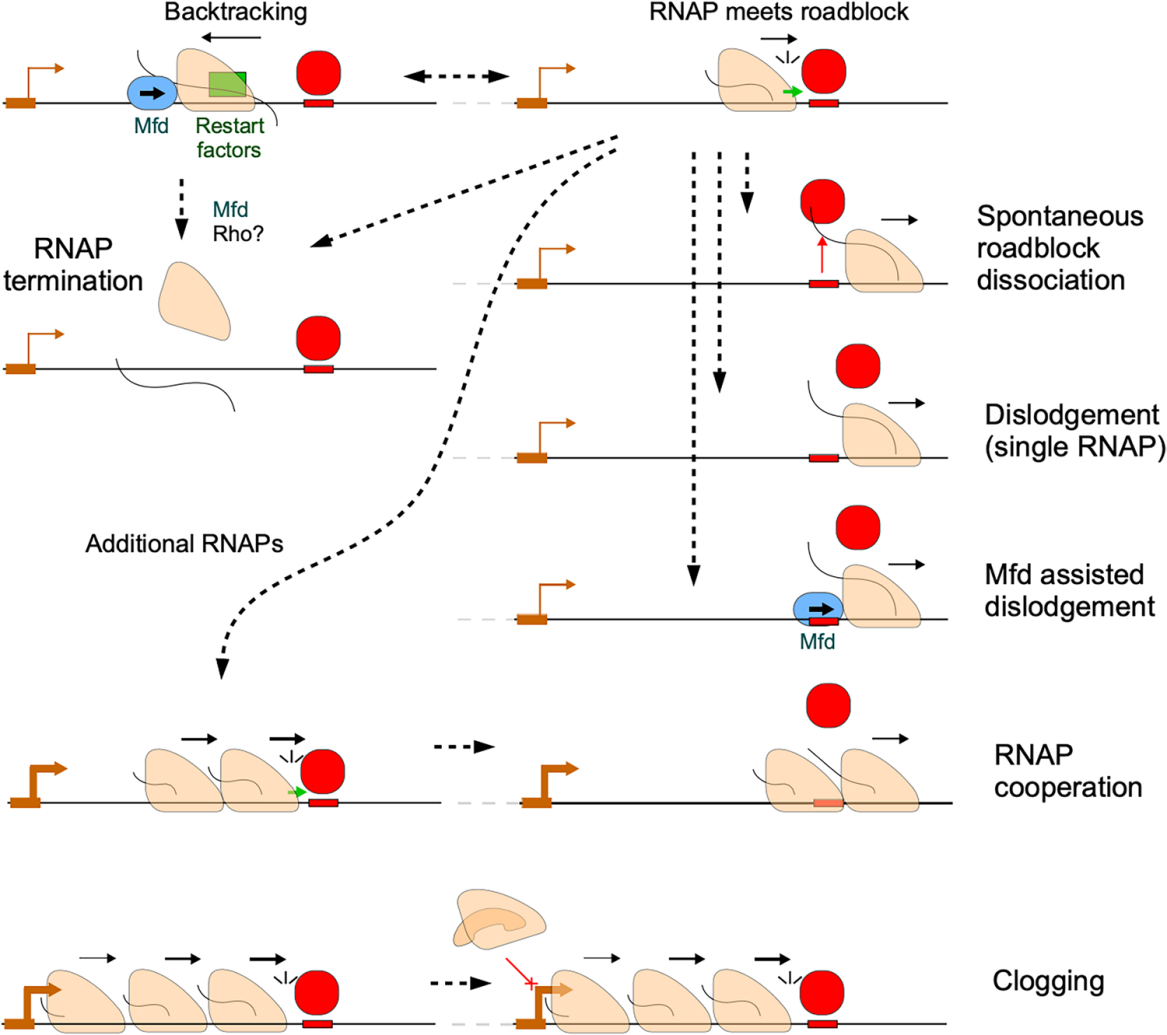
Possible outcomes of RNAP-roadblock interactions. When a roadblock protein (red) binds to the DNA and blocks the progression of an RNAP (tan), the RNAP may backtrack along the DNA. However, specialized proteins such as Mfd (blue) or transcript cleavage factors (green) can help reactivate the backtracked RNAP. If the RNAP is not reactivated, it is subject to termination by Mfd or possibly Rho. Alternatively, RNAP may be able to pass the roadblock as a result of spontaneous (unassisted) dissociation of the roadblock protein. RNAP may also actively dislodge the roadblock protein from the DNA, with or without the assistance of Mfd. Multiple RNAP at a roadblock site generally increase the fractional read-through due to RNAP cooperation, which can either increase the roadblock dislodgement or reduce RNAP backtracking and termination. However, when multiple RNAP build up at a roadblock, the promoter site may become obstructed, blocking further initiation from the promoter, in a process known as clogging

The interactions between elongating RNAP and static protein roadblocks have been extensively studied in bacteria using both *in vitro* and *in vivo* approaches (Hao et al. 2014; He and Zalkin 1992; Lewis et al. 2008; Voros et al. 2017; Xu et al. 2022). In recent years, similar behaviour has also been observed in eukaryotic systems (Colin et al. 2014; Hedouin et al. 2022; Shukla et al. 2011), highlighting the conserved nature of this transcriptional regulatory mechanism in gene regulation. Several factors have been identified that can affect the outcomes of RNAP-roadblock protein interactions (Fig. 1), including the sequence context of the DNA region where the roadblock is located and the binding kinetics of the roadblock protein (Hao et al. 2014, 2016).

When an elongation complex (EC) encounters a roadblock, it may stall and subsequently backtrack along the DNA, causing the 3′ end of the nascent RNA transcript to become displaced from the catalytic site (Fig. 1). This leads to the formation of an inactive RNAP complex that halts further transcription (Nudler 2012; Toulmé et al. 2005). Several mechanisms have evolved to overcome RNAP backtracking (Fig. 1). For example, bacterial transcription factors GreA and GreB can rescue stalled RNAP by cleaving the RNA transcript, generating a new 3′ end at the catalytic site to reinitiate transcription (Abdelkareem et al. 2019; Toulmé et al. 2005; Yuzenkova et al. 2014). Mfd, a bacterial transcription-coupled repair (TCR) factor, can also reactivate a stalled or backtracked RNAP (Borukhov et al. 2005), using its ATP-dependent translocation activity to push the RNAP forward. This allows the 3′ end of the nascent RNA transcript to re-engage at the catalytic site (Park et al. 2002). Once the EC is successfully reactivated, the elongation complex continues transcription, and re-approaches the roadblock (Fig. 1). Several other proteins have also been implicated in preventing EC pausing and backtracking. These include the bacterial transcription factor DksA, which minimises RNAP pausing by reducing nucleotide misincorporation (Roghanian et al. 2015), and the NusG family of transcription factors, which stabilise the minimal transcription bubble (Kang et al. 2018; Turtola and Belogurov 2016). In bacteria, transcription and translation are often coupled, and the ribosome trailing behind the RNAP can exert a "push" force to reactivate transcription of a backtracked polymerase (Nudler 2012; Proshkin et al. 2010). Mathematical modelling suggests that an RNAP trailed by a ribosome is ~ 13 times more resistant to termination than an untrailed RNAP in a head on collision (Hoffmann et al. 2019).

RNAP pausing and backtracking can also occur in eukaryotes as the EC progresses through nucleosomes (Teves et al. 2014) or encounters protein roadblocks (Candelli et al. 2018), and the mechanisms for resolving backtracking are similar to those in bacteria. For example, the eukaryotic NusG family protein Spt4-Spt5 interacts directly with the EC, the upstream DNA, and the nascent transcript near the RNA exit channel to repress RNAP pausing and backtracking (Ehara et al. 2017; Hartzog and Fu 2013; Klein et al. 2011). In addition, RNAP backtracking can also be suppressed by the transcription initiation factor TFIIF, which occurs through transient interactions between TFIIF and RNAP at the pause site, resulting in a conformational change in RNAP (Schweikhard et al. 2014). However, when backtracking is inevitable, mechanisms also exist to resume transcriptional elongation and prevent prolonged RNAP pausing. For instance, the eukaryotic transcriptional factor TFIIS protein can generate a new 3′ end by cleaving the nascent RNA transcript, much like the GreA/GreB proteins in bacteria. This allows the RNA to realign with the active site (Schweikhard et al. 2014). TFIIS can also bind competitively to the backtrack site, displacing and mobilizing RNA, thereby weakening the grip of RNAPII on the backtracked RNA (Cheung and Cramer 2011; Farnung et al. 2022).

One outcome of this cycle of roadblock encounter and backtracking is termination of the RNAP, with the result that downstream transcription is reduced (Fig. 1). We found that Mfd is a major factor in roadblock-induced termination, at least at a strong roadblock, with the absence of Mfd significantly increasing transcription downstream of a LacI roadblock, particularly for weak promoters (Hao et al. 2014). When the EC stalls at the roadblock site for an extended period of time and is unable to overcome the obstacle, Mfd can use its ATPase activity to facilitate the dissociation of RNAP from the DNA, leading to the termination of transcription (Borukhov et al. 2005; Hao et al. 2019; Le et al. 2018). This activity is similar to Mfd’s role in transcription-coupled repair where it removes RNAPs stalled at DNA lesions. Mfd can also remove RNAPs stalled by encounter with a replisome (i.e., a mobile roadblock) that is moving in either the same or opposite direction as the RNAP to mitigate the resultant DNA damage, and genomic instability (Pomerantz and O'Donnell 2010). The bacterial RNA-binding termination factor Rho accounts for approximately 50% of all termination events in *E. coli* (Cardinale et al. 2008), but it remains unclear whether Rho plays a role in terminating RNAP stalled at roadblocks (Dutta et al. 2008; Peters et al. 2011).

The other outcome for the RNAP is to pass through the roadblock (Fig. 1). To do this, RNAP can simply take advantage of the spontaneous dissociation of the roadblock protein and escape a roadblock site before the protein can rebind to the DNA. Alternatively, RNAP may actively remove the roadblock protein from the DNA, a process we term dislodgment (Hao et al. 2014). Mfd’s ability to push RNAP forward may aid dislodgement (Hao et al. 2014), akin to its ability to assist RNAP in traversing difficult-to-transcribe DNA sequences where RNA secondary structure formation at the RNAP exit channel can lead to RNAP stalling (Ragheb et al. 2021).

When a strong promoter is involved, multiple RNAPs can queue up along the DNA upstream of the roadblock. In general, this results in a larger fraction of RNAPs passing the roadblock site, a process termed cooperation (Epshtein and Nudler 2003; Hao et al. 2014; Jin et al. 2010). The mechanism of cooperation is unclear. Trailing RNAPs can prevent backtracking by assisting the translocation of the leading EC, which should increase the number of roadblock-passing attempts. The presence of multiple RNAPs may also inhibit termination, possibly by blocking access by Mfd. Trailing RNAPs may push the leading RNAP to help it dislodge the roadblock protein. In addition, once a leading RNAP passes the roadblock, the following RNAPs may be able to pass before the roadblock protein rebinds or may even occlude the binding site to inhibit its rebinding.

While a high flux of RNAPs from a strong promoter can increase transcription over a protein roadblock, this effect only occurs up to a certain threshold. At very high rates of promoter firing, transcription can decrease due to a build-up of RNAPs, a process known as clogging (Fig. 1). Clogging occurs when backed-up RNAPs occupy the promoter, blocking transcription initiation and reducing the rate of transcription (Sigurdsson et al. 2010; Washburn et al. 2003; Yuzenkova et al. 2014). This phenomenon is more likely to occur when the roadblock is strong, and when the roadblock site is relatively close to the promoter (Epshtein et al. 2003; Hao et al. 2014).

## Natural and engineered protein roadblocks

Only a small number of DNA-binding proteins have been found to be effective roadblocks to transcribing RNAPs. The discovery and study of these proteins has shed light on the important properties of the roadblock proteins that impede the progression of RNAP.

The idea that a static DNA bound complex could block the progression of RNAP downstream of the promoter began with Kassavetis et al. in 1978 who found that RNAP inactivated by rifampicin is unable to initiate a long chain RNA synthesis but retains its ability to bind to promoter sites on the DNA. This then acts as a barrier against other RNAP molecules that have initiated transcription at upstream promoter sites, with the potential to block progression along the DNA (Kassavetis et al. 1978). Similarly, a study by Kingston et al*.* on RNAP pausing within the *E. coli* ribosomal RNA operon found that RNAP bound at a downstream promoter, if slow to initiate, can act as a potential barrier to a second RNAP initiated from an upstream promoter (Kingston and Chamberlin 1981).

After the initial demonstrations that an elongating RNAP can be blocked by other DNA occupants that share the same DNA, one of the first roadblock proteins to be extensively studied was the *E. coli* Lac repressor (LacI). LacI regulates the expression of the *lac* operon by binding to the operator region of the *lac* operon, repressing transcription by blocking the binding of RNAP to the promoter. It also serves as a sensor for lactose, binding to it and causing a conformational change that weakens its affinity for the operator region, thus allowing transcription to occur in the presence of lactose (Jacob and Monod 1961).

In 1982, Horowitz et al. investigated the *lac* and *trp* operons in bacteria that are expressed in opposite orientations in the same region of DNA. They found that LacI bound at the *lacUV5* promoter limited the expression of the *trp* operon by blocking the RNAP initiated at this operon (Horowitz and Platt 1982). A later study found that LacI, when bound at an operator site, can halt the progression of RNAPs and interrupt transcription (Deuschle et al. 1986). The *E. coli* LacI repressor was later also shown to be an effective roadblock for eukaryotic RNAPI and II (Deuschle et al. 1990; Kuhn et al. 1990; Reines and Mote 1993). The ability of LacI to block the progression of RNAP was also demonstrated *in vitro* via single-molecule experiments (Voros et al. 2017; Xu et al. 2022). More recently, our laboratory has used a synthetic biology approach to systematically analyse the LacI roadblocking *in vivo* (Hao et al. 2014), and determined the key parameters of effective roadblocking (see below).

The discovery of the Lac repressor as a transcriptional roadblock has paved the way for the identification of additional roadblock proteins in recent years. By binding downstream of transcription start sites, these proteins have all been shown to block the progression of elongating RNAP. Examples of such proteins include transcription factors like PurR, GalR and ArsR repressors in *E. coli* (He and Zalkin 1992; Lewis et al. 2008; Merulla and van der Meer 2015), CcpA and CodY in *B*. *subtilis* (Choi and Saier 2005), and various other cellular proteins, such as the dominant growth phase nucleoid protein Fis (Chintakayala et al. 2013), Tus replication terminator protein (Guajardo and Sousa 1999), histone-like nucleoid structuring protein H-NS (Dole et al. 2004), and the SOS response protein LexA (Sancar et al. 1982). In addition, a recent study by Klimuk et al. found that the controller protein C of the bacteria Kpn2I restriction-modification system can also function as a roadblock to RNAP, preventing transcription of its own gene and thus forming a negative feedback loop to control its own expression (Klimuk et al. 2018).

The phenomenon of transcriptional roadblocking is not limited to prokaryotic systems. Next-generation sequencing has shown the widespread occurrence of RNAPII pausing and roadblock termination at sites occupied by transcription factors in both yeast and human cells (Candelli et al. 2018; Mayer et al. 2015). For example, CTCF, a zinc finger binding protein capable of sequence-specific binding to specific DNA sequences known as insulators, was found to pause RNAPII in a mammalian model system (Kornblihtt 2012; Shukla et al. 2011). Similarly, the yeast general regulatory factor Reb1p and repressor activator protein Rap1p have both been shown to block RNAPII progression when bound at their high affinity sites (Colin et al. 2014; Roy and Chanfreau 2018; Yarrington et al. 2012). Notably, the ability of Rap1p to block RNAPII is dependent on its binding orientation, as binding in the inverted orientation can render it susceptible to dislodgement by RNAPII (Yarrington et al. 2012). It has been proposed that the interaction between the C terminus of the Rap1p DNA binding domain and its DNA binding site creates additional contacts, reducing the likelihood of its displacement by RNAP. Finally, the yeast Cbf1 protein was recently shown to serve as a transcriptional roadblock, preventing unscheduled transcription from entering the centromeres and thus safeguarding these critical regions (Hedouin et al. 2022).

Apart from naturally occurring roadblock proteins, several proteins have also been engineered to function as transcriptional roadblocks. One of the first examples is EcoRI Q111, a mutant version of the EcoRI endonuclease that has had its DNA cleavage activity removed via targeted mutagenesis. This modified EcoRI retains its DNA binding site affinity and effectively blocks RNAP progression *in vitro* (Pavco and Steege 1990). Another example of an engineered roadblock protein is the TALE proteins, which are DNA binding proteins found in *Xanthomonas* bacteria. TALE proteins recognize specific DNA sequences through repeating units of 33–35 highly conserved amino acids, including two critical amino acids known as repeat variable di-residues (RVDs). By modifying the RVD sequence, TALE proteins have been engineered to bind a *lac* operator with high affinity, resulting in a more potent roadblock than the Lac repressor (Politz et al. 2013). The catalytically dead CRISPR-Cas (dCas) protein is another example of an engineered roadblock protein, which will be discussed in more detail below.

## Programmable roadblocks by catalytically dead CRISPR-Cas proteins

In the last decade, the rapid development of CRISPR-Cas (clustered regularly interspaced short palindromic repeats-CRISPR associated) technology, derived from the adaptive immunity system found in a large number of bacteria and archaea (Barrangou et al. 2007; Makarova et al. 2006), has been fundamental in the expansion of genome editing in a variety of organisms, including bacteria, plants, animals, and human cells (Anzalone et al. 2020; Cong et al. 2013; Doudna and Charpentier 2014; Makarova et al. 2015; Mali et al. 2013).

One of the best studied CRISPR-Cas systems is the type-II system, which consists of a single multidomain effector protein known as Cas9. Cas9 can cut the DNA at specific sites designated by a single guide RNA (sgRNA) that is complementary to the target DNA sequence (Gasiunas et al. 2012; Jinek et al. 2012; Wiedenheft et al. 2012). Once the DNA is cut, the natural repair mechanisms of the cell are triggered to generate indel mutations, effectively changing the target DNA sequence. However, for Cas9 to bind and cut DNA, a PAM (Protospacer Adjacent Motif) sequence must be present adjacent to the target DNA sequence (Hille et al. 2018). Different Cas9 proteins have different PAM requirements, and this PAM specificity is crucial for minimizing off-target effects and ensuring precise genome editing. The widely used Cas9 from *Streptococcus pyogenes* requires an NGG PAM, which restricts the sequence space that is targetable by Cas9. However, structure-guided rational design and directed evolution have been used to significantly broaden the target range of *Sp* Cas9 (Hu et al. 2018; Kleinstiver et al. 2015; Nishimasu et al. 2018; Walton et al. 2020), thereby enabling genome editing of regions previously inaccessible to *Sp* Cas9.

While Cas9 is a highly efficient DNA-cutting enzyme, its ability to bind to DNA independently of its nuclease activity has also led to the development of catalytically inactive Cas9, known as dCas9 (Jinek et al. 2012). This mutant version of Cas9 has been used as a versatile molecular scaffold to bring various effectors, such as transcriptional modulators and epigenetic modifiers, to targeted DNA locations (Dominguez et al. 2016; Gilbert et al. 2013). In addition, a number of studies have demonstrated that dCas9 itself can also regulate gene expression in prokaryotes by blocking the progression of RNAPs (Bikard et al. 2013; Peters et al. 2016; Qi et al. 2013; Vigouroux et al. 2018; Widom et al. 2019). Similar to yeast Rap1p protein, the roadblocking activity of dCas9 is dependent on its binding orientation. Effective repression of transcription elongation is observed when targeting the non-template strand, whereas less effective repression is observed when targeting the template strand (Bikard et al. 2013; Hao et al. 2016; Qi et al. 2013). Interestingly, while a DNA-bound dCas9 can also impede the progress of the replisome, this roadblocking activity is not dependent on binding orientation (Doi et al. 2021; Whinn et al. 2019).

The location of dCas9 targeting sites relative to the transcriptional start site is also found to be important for effective roadblocking. Target regions located further from the promoter resulted in less effective roadblocking, possibly due to the extended sequence allowing for multiple RNAPs to build up and use the cooperation effect to ‘push back’ against backtracking or ‘push’ past a bound roadblock. The roadblocking effect is also reduced at a target region very close to the promoter site, but it can still reduce promoter activity either by blocking the promoter site for RNAP initial binding or resulting in a clogging effect from a build-up of RNAP (Bikard et al. 2013).

In addition to RNAP roadblocking, dCas9 can also reduce the expression of a target gene by targeting the promoter sequence to directly block RNAP binding at the promoter. This repression however differs from roadblocking and is not dependent on the orientation of Cas9 binding (Bikard et al. 2013; Qi et al. 2013).

More recent studies of *Sp* dCas9 have been able to tune dCas9 roadblocking to allow for precise and robust changes in targeted gene expression by controlling complementarity between the guide RNA and the target DNA sequence through the introduction of mismatches in the 20nt sgRNA sequence (Vigouroux et al. 2018). Several studies have found that mutations within the last 6–8 nucleotides at the PAM-distal end of the sgRNA did not have a significant impact on the roadblock activity of dCas9, but mutations within the 7nt at the PAM-proximal end almost completely abolish the dCas9 roadblocking (Fu et al. 2013; Jinek et al. 2012; Widom et al. 2019). It is unclear how much these effects can be explained by reduced occupancy of the site by dCas9 or by increased dislodgement of bound dCas9 by RNAP. Recent structural studies have shown that PAM-distal mismatches can be accommodated either by base skipping and multiple noncanonical base pairing, or by stabilization through reorganization of the RuvC nuclease domain (Bravo et al. 2022; Pacesa et al. 2022). These findings provide new insights for designing better Cas9 variants with increased fidelity.

Other Cas9 orthologs have also demonstrated dCas9 mediated roadblocking, such as *Streptococcus thermophilus* CRISPR1 and *Neisseria meningitidis* Cas9 proteins. These orthologs have varying PAM requirements, providing a wider range of potential target sites (Esvelt et al. 2013). More recently, a bioinformatic screen has identified 79 phylogenetically distinct Cas9 orthologs, along with their corresponding gRNA and PAM requirements (Gasiunas et al. 2020). It is likely that many of these Cas9 proteins, when mutated to remove their catalytic activity, will act as roadblocks to elongating RNAPs.

**Fig. 2:**
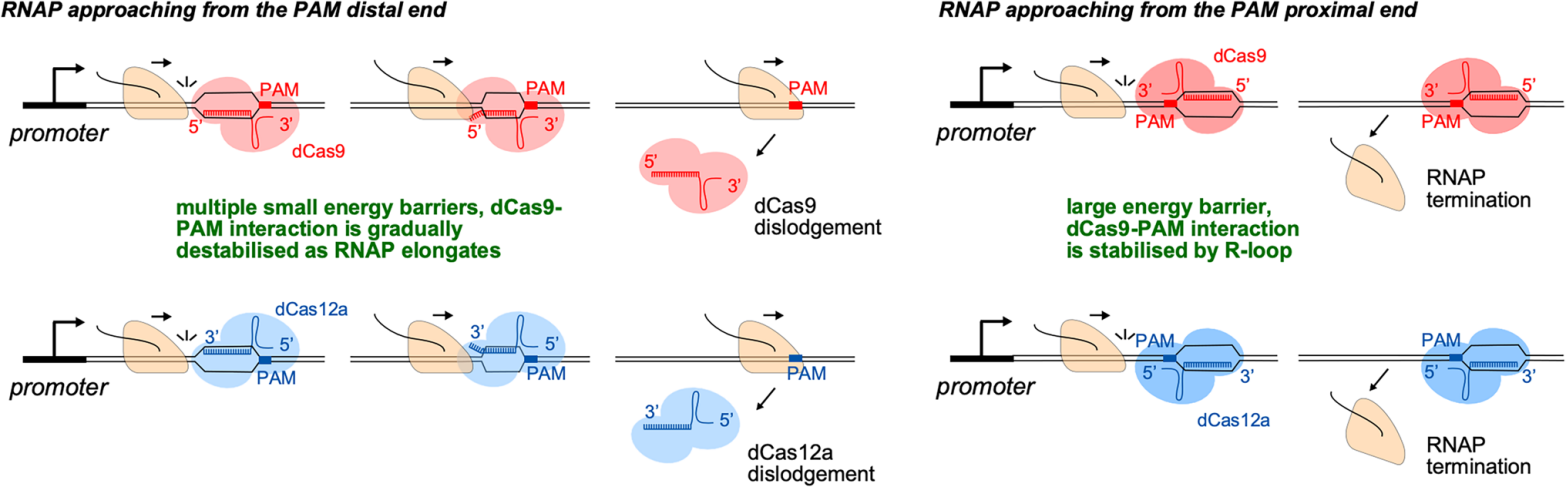
Model for the strand-dependent roadblocking by the dCas9 and dCas12a complexes. As RNAP moves forward towards the dCas complex from the PAM-distal end, it weakens the numerous small energetic barriers that form between the guide RNA and DNA. With each step, the dCas-PAM interaction becomes slightly destabilized. As a result, by the time RNAP reaches the PAM end, the final barrier is weak and easily overcome, enabling RNAP to dislodge the dCas complex. Conversely, the dCas-PAM interaction at the PAM-proximal end is fully stabilized by the R-loop, creating a large energetic barrier that RNAP must overcome in a single step

Similar to Cas9, other type-II CRISPR associated single effector proteins from various bacteria are known to be guided to a target site complementary to a region within the RNA guide to cleave a target (Makarova et al. 2015). One such protein is Cas12a (previously known as Cpf1), a single-RNA guided endonuclease from the type-II V CRISPR system that, in contrast to Cas9, requires the presence of an upstream T-rich PAM sequence (Fig. 2) and produces a staggered double stranded break in the DNA (Leenay et al. 2016). A catalytically dead version of Cas12a (dCas12a) has been shown to function as a transcription roadblock (Zetsche et al. 2015) with a strand dependence opposite to that of dCas9 (Fig. 2) (Miao et al. 2019). However, the experiments were conducted using different guide RNAs that target either the template or non-template strand of DNA. It is possible that the differences in roadblocking efficiencies could be due in part to the varying binding affinities of different guide RNA/dCas complexes to DNA. An ideal experiment would be to use the same guide RNA to target the identical DNA sequence in both orientations.

A recent single-molecule study has shed light on the mechanism of orientation-dependent roadblocking by dCas9 and dCas12a (Hall et al. 2022). The DNA-bound dCas complex consists of two distinct regions: an unprotected R-loop-mediated DNA bubble located on the PAM-distal end and tightly clamped DNA located on the PAM-proximal end (Jinek et al. 2014; Stella et al. 2017; Swarts et al. 2017). According to the model of Hall et al., when RNAP approaches the dCas complex from the PAM-distal end, forward translocation forces the re-zipping of the DNA bubble in the dCas complex. This causes the R-loop to collapse and dCas to detach from the DNA, resulting in more permissive transcription from the PAM-distal side. Conversely, when RNAP approaches a bound dCas from the PAM-proximal side, the R-loop in the Cas complex is not readily accessible to the elongating RNAP, resulting in more prohibitive transcription from the PAM-proximal side. In line with this model, a modified sgRNA that can stabilise the R-loop enhances the effectiveness of the dCas9 roadblock when RNAP approaches from the PAM-distal side (Hall et al. 2022). We expect that the positive supercoiling generated ahead of RNAP may also aid R-loop collapse. Importantly, when approaching from the bubble side, each RNAP elongation step need only remove one RNA–DNA basepair. Thus, RNAP encounters a series of small energetic barriers that it can easily overcome one by one. Each step slightly destabilizes the dCas-PAM interaction, so that once RNAP reaches the PAM, the final barrier is very weak. In contrast, when RNAP approaches from the other direction, the dCas-PAM interaction is fully stabilized by the R-loop and may be required to be removed in one step, thus presenting a large energetic barrier (Fig. 2).

## What makes a protein a strong or a weak roadblock?

While several DNA-binding proteins have been identified as roadblocks, the majority of DNA binding proteins are not effective transcriptional roadblocks. For example, neither the CI lysogenic repressor nor the Cro repressor of bacteriophage λ bound to their three-operator OR site is a strong roadblock (Hao et al. 2016). Our laboratory has also shown poor roadblocking by the CII activator protein of phage λ (Palmer et al. 2009) and the CI repressor (Shearwin et al. 2002) and CII activator proteins of phage 186 (Hao et al. 2019; Neufing et al. 2001). The inability of the phage CI repressors to block RNAP progression was also shown in a recent in vitro single-molecule study (Lu et al. 2022). This raises questions about what properties of proteins cause them to be strong roadblocks to RNAP.

To understand the key parameters that govern transcriptional roadblocking, we performed systematic experiments combined with modelling to study LacI roadblocking *in vivo* (Hao et al. 2014). We varied the promoter firing rate (15 strengths from 0.00033 to 0.2 sec^−1^), the LacI concentration (5 levels from 15 to 254 nM), operator affinity (3 strengths from 0.17 to 3.9 nM), the rate of RNAP termination (wild type and *mfd* knockout), and promoter–roadblock spacing (3 spacings from 30 to 102 base pairs) and measured the magnitude of roadblocking. To dissect the complex interaction of these factors, we utilized stochastic simulations.

**Fig. 3:**
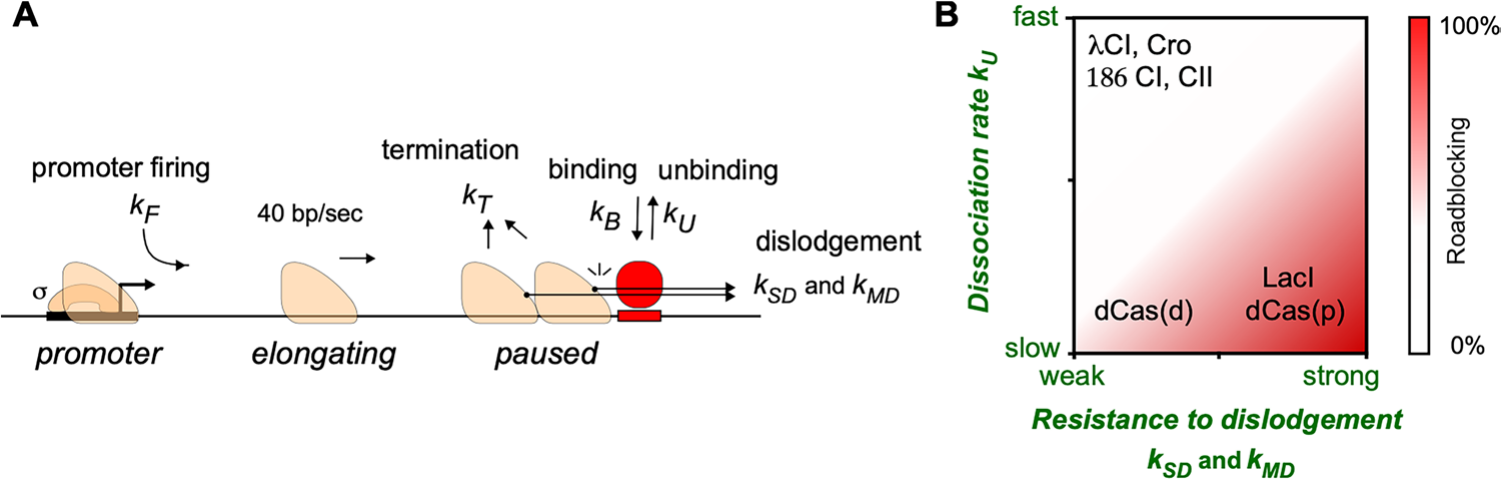
Biophysical determinants of effective transcriptional roadblocking. **A**) A stochastic model of transcriptional roadblocking indicating the key parameters that govern transcriptional roadblocking. k_SD_ and k_MD_ are the rates of roadblock dislodgement by single or multiple RNAPs, respectively. Figure adapted from Fig. 1C in (Hao et al. 2014). **B**) The kinetic properties of a DNA-binding protein and its resistance to dislodgement by an elongating RNAP collectively determine its ability to effectively block RNAP progression. λCI, Cro: the CI and Cro repressors from bacteriophage λ; 186 CI, CII: the CI repressor and CII activator of bacteriophage 186; dCas(d): an RNAP approaching dCas complex from a PAM-distal end; dCas(p): an RNAP approaching dCas complex from a PAM-proximal end. Figure adapted from Fig. 2A in (Hao et al. 2017)

We used a hybrid Gillespie/discrete fixed time-step method. In the absence of RNAP on the DNA, only three possible events can occur: loading of a new RNAP at the promoter (if there is no other RNAP blocking it), and binding or unbinding of LacI, depending on whether the operator is occupied. The next time for each of these processes was determined by the formula ‘– ln(*r/k*)’, where r is a random number between 0 and 1 and *k* (s^−1^) is the rate of the process (Fig. 3). The process with the next shortest time was used to update the system, and the time was incremented by the time associated with that process. This simulation procedure was repeated until an RNAP was loaded on the DNA, at which point the simulation was switched to a fixed time-step mode. In this approach, each step was set to the time taken for RNAP to move forward one base pair (1/40 s), and in each time step, each RNAP attempted to advance 1 bp. All events were assigned a specific rate *k* (Fig. 3). The occurrence of any particular event, if possible, during the next time step was determined by generating a random number between 0 and 1; if this number was less than 1- e^–*k*^, then that event occurred. After all RNAPs were cleared from the DNA, the simulation was switched back to the Gillespie approach.

The events that are included in the simulation are as follows (Fig. 3A):

1. *Promoter firing*: Binding and initiation of transcription by RNAP is simulated as a single-step process with a rate *k*_*F*_.
2. *Binding and unbinding of the roadblock protein*: If the operator is unoccupied by either another Lac repressor or elongating RNAPs, the rate of binding of LacI (*k*_*B*_) is influenced by the concentration of LacI and is determined by the product of the binding constant (*k*_*on*_, s^−1^ M^−1^) and its concentration. On the other hand, the rate of unbinding (*k*_*U*_, s^−1^) is dependent on the specific operator sequence.
3. *Elongation*: The simulation begins by attempting to move each RNAP forward by 1 bp, starting from the RNAP farthest from the promoter. If an RNAP encounters a roadblock, caused by either LacI or a paused RNAP, it can move forward only if it or the leading RNAP is capable of dislodging LacI. The dislodgement rate by a single RNAP is *k*_*SD*_. If multiple RNAPs are queued in front of a roadblock, a different dislodgement rate (*k*_*MD*_) is applied to account for RNAP cooperation. Importantly, we found that this active dislodgement of the roadblock by RNAP was needed to reproduce the experimental data. A successful dislodgement of LacI was taken to allow all RNAPs queued at the roadblock to advance. Once an RNAP moves past the last position of the operator, a new transcript is counted, and that RNAP is removed from the DNA.
4. *RNAP Termination*: Each paused RNAP can be removed from the system with rate *k*_*T*_.

Our modelling highlighted protein kinetics as the key determinant for transcription roadblocking. To be a good roadblock, the protein’s rate of binding must be sufficiently in excess of its unbinding rate to ensure that its fractional occupancy of the roadblock site is high (fractional occupancy = *k*_*B*_/(*k*_*B*_ + *k*_*U*_)). However, *k*_*B*_ and *k*_*U*_ affect roadblocking in different ways. The rate of binding *k*_*B*_ has minimal impact on roadblocking unless the promoter is very strong, whereas the rate of unbinding of the roadblock protein *k*_*U*_ is critical. If unbinding is rapid, then there is a greater chance that the roadblock protein will spontaneously dissociate before RNAPs stalled at the roadblock are terminated. A low *k*_*U*_ implies a higher energy barrier for the protein to leave its site, which should also cause it to be more resistant to active dislodgement by RNAP. Indeed, we found that the fitted rates for dislodgement by single and multiple RNAPs (*k*_*SD*_ and *k*_*MD*_) were inversely correlated with *k*_*U*_ for the different Lac operators.

Consequently, roadblocking and site occupancy can be adjusted independently to some extent, and proteins with the same fractional occupation of a site, but with distinct kinetics, can have different roadblocking properties. Proteins with slow kinetics (low *k*_*B*_, low *k*_*U*_ e.g. LacI), will tend to be strong roadblocks, while proteins with fast binding kinetics (high *k*_*B*_, high *k*_*U*_ e.g. λ CI) will tend to be ineffective roadblocks (Fig. 3B).

However, not all proteins with slow binding kinetics are strong roadblocks to elongating RNAP, as demonstrated by dCas9 (Bikard et al. 2013; Hao et al. 2016; Qi et al. 2013)(Fig. 3B). The strong orientation bias for roadblocking cannot be simply explained by binding kinetics because the values of *k*_*B*_ and *k*_*U*_ for dCas9 are the same, regardless of the direction RNAP approaches from. This could be accounted for in our model by setting different active dislodgement rates for the two directions, thus breaking the correlation between *k*_*U*_ and the rates for *k*_*SD*_ and *k*_*MD*_. We expect that a more complete model of dCas9 roadblocking would require incorporating multistep unbinding.

Another factor that can affect roadblocking is DNA looping, with weak roadblocks strengthened when participating in long-range interactions on the DNA. For example, a single-molecule study by Voros et al. demonstrated that the LacI repressor can act as a robust roadblock even when bound to a weak lacO2 operator, as long as it participates in a DNA loop to another LacI site (Voros et al. 2017). Similarly, our study on the λ CI repressor found that CI binding in a 3.8 kb loop with the OL binding site improved CI roadblocking at the OR site *in vivo* (Hao et al. 2016). It is not clear whether these effects can be explained by the expected increased binding rate provided by DNA looping or if the spontaneous or RNAP-assisted unbinding rates are affected. We have recently developed a quantitative *in vivo* assay to measure the looping efficiencies of various natural and engineered proteins (Hao et al. 2021) that should help understand the mechanism of this effect.

## Conclusion and future perspective

With recent advances in high-throughput RNA sequencing techniques, transcriptional roadblocking has increasingly been recognized as an important control mechanism in gene regulation in both bacteria and eukaryotic cells. Roadblock-mediated termination has been shown to prevent uncontrolled gene expression in *E. coli* (Chintakayala et al. 2013), as well as protect the yeast centromere from off-target transcription (Hedouin et al. 2022). Additionally, roadblock proteins have been suggested to work in tandem with the cellular canonical termination pathways to delimit transcription units and reduce pervasive transcription (Candelli et al. 2018).

In this review, we summarised the mechanisms by which roadblock proteins impede the progression of elongating RNAP and the cellular factors that can facilitate RNAP escape or clearance of roadblocks. We surveyed the literature on factors that influence a DNA-bound protein's effectiveness as a roadblock and identified protein binding dynamics and resistance to RNAP dislodgement as key defining factors for a strong roadblock. Further investigation into the molecular regulation of roadblocking and identification of key attributes for effective roadblocks would significantly enhance our understanding of gene regulation and potentially open new avenues for designing synthetic genetic circuits and therapeutic interventions.

For example, roadblocking regulation has been used to build an arsenic biosensor with ultra-low background and high sensitivity, based on the *E. coli* arsenite-responsive ArsR repressor (Merulla and van der Meer 2015). Likewise, Chatterjee and colleagues used LacI roadblocking and transcriptional interference (TI) to construct a variety of synthetic two-input logic gates and investigated the factors that influence their behaviour (Bordoy et al. 2019; O'Connor et al. 2021).

Our own research on transcriptional interference between convergent promoters also revealed that transcription factors can indirectly control a target promoter by regulating the interfering promoter (Hao et al. 2016, 2017). This approach is widely used in developmental switches across all kingdoms of life (Dodd et al. 2007; Hongay et al. 2006; Latos et al. 2012) to ensure mutual exclusivity between different developmental programs. However, for this strategy to be effective, the transcription factor must have rapid binding kinetics to maintain site occupancy without blocking RNA polymerase.

Recent discoveries in the use of catalytically dead Cas proteins as programmable roadblocks to RNAP, with the potency of dCas roadblocking that is adjustable through targeting different DNA strands and through guide RNA mismatches, have opened up exciting opportunities to uncover novel aspects of biology. Genome-wide dCas9 knockdown (CRISPRi) identified essential genes in *E. coli*, including a subset that are highly sensitive to minor expression fluctuations via roadblocking with less effective template targeting (Rousset et al. 2018). Additionally, CRISPRi has been utilized to identify genes required for phage infection by 14 phylogenetically diverse phages (Mutalik et al. 2020; Rousset et al. 2018).

## Data Availability

The article type (review article) does not expect raw files.
